# M2M Communication Assessment in Energy-Harvesting and Wake-Up Radio Assisted Scenarios Using Practical Components

**DOI:** 10.3390/s18113992

**Published:** 2018-11-16

**Authors:** Jukka Rinne, Jari Keskinen, Paul R. Berger, Donald Lupo, Mikko Valkama

**Affiliations:** 1 Lab. of Electronics and Communications Engineering, Tampere University of Technology, FI-33720 Tampere, Finland; jari.keskinen@tut.fi (J.K.); paul.berger@tut.fi (P.R.B.); donald.lupo@tut.fi (D.L.); mikko.e.valkama@tut.fi (M.V.); 2 205 Dreese Laboratory, Department of Electrical and Computer Engineering, The Ohio State University, 2015 Neil Avenue, Columbus, OH 43210-1272, USA

**Keywords:** wireless energy harvesting, M2M communications, wake-up radio, Shannon limit, propagation loss, diversity system, supercapacitor, perpetual communications

## Abstract

Techniques for wireless energy harvesting (WEH) are emerging as a fascinating set of solutions to extend the lifetime of energy-constrained wireless networks, and are commonly regarded as a key functional technique for almost perpetual communications. For example, with WEH technology, wireless devices are able to harvest energy from different light sources or Radio Frequency (RF) signals broadcast by ambient or dedicated wireless transmitters to support their operation and communications capabilities. WEH technology will have increasingly wider range of use in upcoming applications such as wireless sensor networks, Machine-to-Machine (M2M) communications, and the Internet of Things. In this paper, the usability and fundamental limits of joint RF and solar cell or photovoltaic harvesting based M2M communication systems are studied and presented. The derived theoretical bounds are in essence based on the Shannon capacity theorem, combined with selected propagation loss models, assumed additional link nonidealities, diversity processing, as well as the given energy harvesting and storage capabilities. Fundamental performance limits and available capacity of the communicating link are derived and analyzed, together with extensive numerical results evaluated in different practical scenarios, including realistic implementation losses and state-of-the-art printed supercapacitor performance figures with voltage doubler-based voltage regulator. In particular, low power sensor type communication applications using passive and semi-passive wake-up radio (WuR) are addressed in the study. The presented analysis principles and results establish clear feasibility regions and performance bounds for wireless energy harvesting based low rate M2M communications in the future IoT networks.

## 1. Introduction

Advances in technology have made it possible to implement cost-effective Machine-to-Machine (M2M) communications, wireless sensor network (WSN)-based automation, monitoring and control systems [1], where M2M communications is one key technical enabler to many IoT applications (M2M-driven IoT). Internet-of-Things (IoT) is a paradigm of wireless technology, where smart sensors and machines communicate through combining multiple protocols and devices such as Radio Frequency Identification (RFID) and WSN. IoT systems require sensing, gathering, storing, processing and transmitting of data from real time sensors as well as virtual online sensors.

Wireless sensor networks can be used for various applications including home automation, health monitoring, factory automation, process control, real-time monitoring of machinery, monitoring environment, and real-time inventory management. In these systems, sensor nodes monitor and gather the parameters critical to automation processes and transmit the data to host, i.e., a user, a control center or an operator. Since sensor nodes are commonly battery-powered devices, their operational lifetimes are limited. Energy harvesting techniques have thus a good potential to solve this constraint with well designed operational scheduling to use the energy efficiently. It has been recently predicted in [2], that by 2023, there will be tens of billions (31.6 billion) connected devices which should all operate and integrate smoothly with the Internet, while providing a vast spectrum of services, e.g., healthcare, smart homes, industry automation, and environmental monitoring. This trend, commonly referred to as the Internet-of-Things (IoT), Internet-of-Everything (IoE) or Industrial Internet-of-Things (IIoT), imposes enormous challenges and requirements on the radio connectivity, in the form of M2M communications, from coverage, energy-efficiency and scalability points of view [3]. Another closely related field is low-energy sensor networks and energy-harvesting, where the sensor and communication nodes are autonomously extracting or harvesting energy from their surroundings [4,5]. Typically energy-autonomous M2M communications with fairly low bitrates but massive numbers of devices pose substantial demands on the component, circuit, and system designs.

The technological challenges that are under intensive research include low power consumption of the devices [6], and the methods for obtaining or harvesting energy efficiently from different sources, as well as storing the harvested energy for later use [7,8,9,10,11]. It is worth noting, that the energy efficiency may be improved by applying energy optimization methods based on, for example, coalition formation with QoS knowledge [12,13] or data-aggregation [14]. Thorough study on optimization of energy efficient resource allocation in M2M communications with energy harvesting [15] attempts to minimize the total energy consumption of the network via jointly controlling power and time allocation while taking into account circuit power consumption as well as potential QoS and latency constraints. In addition to the basic silicon or CMOS based circuits, also alternative organic/inorganic or printed electronics based solutions are raising interest [16,17,18]. Organic (carbon based) electronics incorporate attractive properties of organic small molecule conductors including their electrical conductivity that can be varied by the concentrations of dopants. These potentially low cost and low carbon footprint solutions may be mechanically flexible and some have high thermal stability [19], which extend their usability in various applications. In essence, with organic electronics there is another area of sustainability that is also critical. It has to do with gathering and using ambient energy or harvesting small amount of energy that allows the systems to be unplugged from the power grid entirely. In the bigger picture, the results of such work will allow the ubiquitous electronics of the future to be manufactured and used in a sustainable way by enabling energy autonomy without the use of toxic materials and by enabling less resource-intensive ways to manufacture electronics. In this work, specifically, the usability limits of aqueous supercapacitors will be evaluated for communication purposes with relatively simple and well known assisting schemes.The contributions of the paper is (a) construction of the realistic modeling environment to be used in evaluations (b) development of the new capacitor model with leakage current with respect to capacitance and recharging scenario, (c) calculation of performance limits using selected model with realistic parameters parameters, and (d) drawing relevant technical conclusions on the feasibility of the overall system incorporating energy-harvesting, energy-storage and wake-up radio based communications.

First, we study the limitations and possibilities of communications with existing technology restrictions. Furthermore, the feasibility and fundamental limits of energy harvesting based machine communication systems are studied and presented. In the study, we adopt fundamental Shannon capacity laws combined with appropriate propagation loss models and assumed levels of nonidealities related to the radio link implementation, to extract fundamental performance bounds and feasibility limits for low-rate low-energy M2M communications. The study also incorporates energy harvesting issues together with the energy storage model in the form of a supercapacitor [20] and selected harvesting methods. We also derive expressions for the available communication distance depending on the energy harvested and storage capabilities, combined with the targeted instantaneous communication rate and the assumed probability to transmit or receive at a given time window. In addition we use also passive and semi-passive wake-up radio concept to enhance the energy efficiency. Diversity receiver principle is included with proper practicality mimicking parameterization. In the numerical evaluations, we specifically focus on the license-exempt ISM bands at 433 MHz and 900 MHz (sub-1 GHz), while the analysis methodology and derived expressions are valid at all other frequencies as well. The provided analysis methodology and obtained results establish clear feasibility regions and performance bounds for energy harvesting based low-rate M2M communications using non-CMOS harvesting technologies with our newly developed organic supercapacitor.

The remainder of this paper is structured as follows. First, in Section 2, the fundamental channel capacity aspects are addressed and discussed [21]. Then, in Section 3, the RF and solar energy harvesting and storage issues are introduced, together with the capacitor recharging topics with harvested power and with operational time of transceiver applying probabilities to transmit and receive. Here also different wake-up receiver issues are addressed. In Section 4, the considered path loss models are first reviewed, followed by an extensive set of numerical results assuming state-of-the-art organic or printed energy harvesting and storage techniques. Finally, the key findings and conclusions are drawn in Section 5.

## 2. Fundamental Limits on Capacity

The theoretical maximum information transfer rate of any noisy channel is given by the Shannon capacity law [22]. As is very well known, this Shannon limit for communication, *R*, can be expressed in bits/s as

(1) $$R=B\log_{2}\left(1+S/N\right),$$

where *B* denotes the bandwidth, *S* refers to the received useful signal power while the noise power is denoted by *N*. At operational frequencies that are higher than 300 MHz, the noise is due to thermal noise [23]. In this case, the power of the noise is given by N=kTBF, where *k*, *T*, *B*, and *F* are the Boltzmann coefficient ($1.3807\times 10^{-23}$ J/K), temperature in Kelvins, bandwidth in Hz, and noise figure (NF) in numeric form ($F=10^{\mathrm{NF}/10}$, where NF is in decibels), respectively. The corresponding capacity in bits/s/Hz can be then expressed as

(2) $$C=R/B=\log_{2}\left(1+\frac{S}{kTBF}\right).$$

By incorporating practical parameters [21], the capacity may be written as

(3) $$C=\log_{2}\left(1+\frac{S_{TX}/\lambda }{\mu kTBF}\right),$$

where $S_{TX}$, λ, and μ, are the transmission power, path loss, and implementation loss factor (≥1), respectively. It is worth noticing that when $M_{d}$-fold diversity is applied with Maximal Ratio Combining (MRC) [24], the capacity may be expressed as

(4) $$C=\log_{2}\left(1+\frac{M_{d}\times S_{TX}/\lambda }{\mu kTBF}\right),$$

where perfect channel state information is assumed to be known. We assume that the path loss can be given by

(5) $$\lambda =d_{1}d^{d_{2}},$$

where $d_{1}$ and $d_{2}$ are parameters related to channel model and are elaborated more later in the manuscript. With the corresponding loss in decibels expressed as

(6) $$\lambda_{\mathrm{dB}}=10\log_{10}\left(d_{1}\right)+10d_{2}\log_{10}\left(d\right),$$

the maximum distance for given capacity requirement can be expressed as

(7) $$d={\left(\frac{M_{d}\times S_{TX}}{d_{1}\times \mu kTBF\times \left(2^{C}-1\right)}\right)}^{1/d_{2}}.$$

The required transmission power for given capacity and operational distance will be used in the performance evaluation later on, when also other additional aspects are addressed more thoroughly in this paper, in Section 4.

## 3. RF and Photovoltaic Energy Harvesting

Energy harvesting applying diverse methods [25,26,27,28,29] like RF energy harvesting, solar energy harvesting, thermal energy harvesting allows almost perpetual use of devices and hence helps in maintaining very large systems as, e.g., batteries are not needed to be maintained or changed regularly.

**Figure 1:**
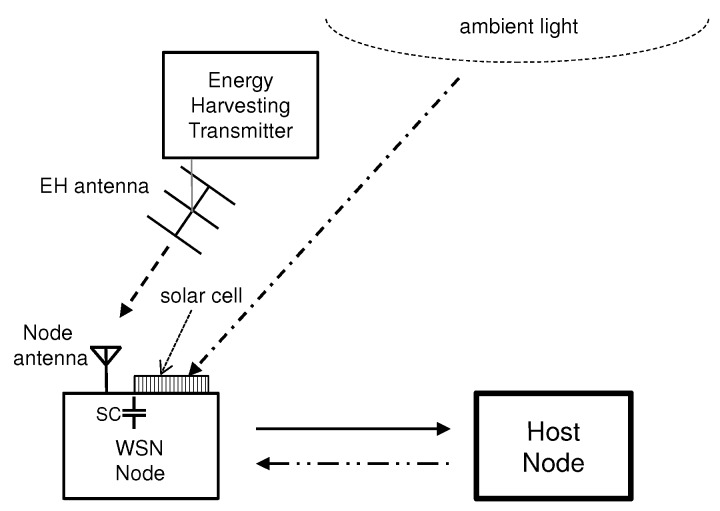
RF energy harvesting link (- - -) and photoenergy harvesting link (- . - . -) between energy sources and WSN node harvesting systems are illustrated. Link between WSN node and Host node (—) and return link between Host node and WSN node (- .. - .. -) are viewed along with supercapacitor (SC).

Scavenging energy is feasible, e.g., from dedicated RF energy harvesting transmitter and solar cell as is shown in Figure 1. The harvested energy is then used to operate energy harvesting transceiver (WSN Node) and communication link between energy harvesting transceiver and receiver (Host Node) can be established. In addition, the Host Node may harvest the energy from the energy harvesting transmitter and ambient light. Thus, the energy sources provide essentially efficient operational assets for large number of devices within their range. Typically, the possible excess harvested energy by the transceiver is stored for later use.

When the harvesting transmitter has transmit power of $P_{htx}$ and the path loss between harvesting transceiver and harvesting transmitter is $\lambda_{h}$, the harvested RF power can be given by $P_{h}^{r}$,

(8) $$P_{h}^{r}=G_{h}\eta_{h}P_{htx}/\lambda_{h},$$

where the gain of the harvesting antenna is $G_{h}$, and efficiency of harvester is $\eta_{h}$, which is typically 0.4 (40%) with current technologies [11]. This power is then usable for transceiver operation and could be also used to maintain its energy storage such as the considered supercapacitor in this paper.

For the photovoltaic power scavenging, the amount of harvested energy, $E_{s}$, depends on light irradiance level, *E*, and solar cell efficiency $\eta_{c}$ as

(9) $$E_{s}=E\eta_{c}.$$

Typically for conventional luminaires, e.g., 100 lx corresponds to 1 W/m${}^{2}$ [30]. For the state-of-the-art organic solar cell [31] the efficiency is within the range of 3% ... 10% ($\eta_{c}=$ 0.03 ... 0.10) and the obtainable powers are 6 μW/cm${}^{2}$ ... 75 μW/cm${}^{2}$ (−22 dBm/cm${}^{2}$ ... −11 dBm/cm${}^{2}$). Thus, the harvested total solar power, $P_{h}^{s}$, using solar cell with surface area, *A*, may be expressed as

(10) $$P_{h}^{s}=E_{s}A,$$

which can be used for transceiver operation and again partly for maintaining its energy storage, when needed. The total harvested power in the system is then $P_{h}=P_{h}^{r}+P_{h}^{s}$.

### 3.1. Energy Storage

In the following, we consider supercapacitor as energy storage unit due to its good cycle life compared to secondary batteries and high energy density compared to traditional capacitors. Assuming that the total power consumed for communication purposes is denoted by $P_{tot}$, it might be so that, $P_{h}<P_{tot}$. That is, more power will be consumed than stored. On the other hand, if $P_{h}>P_{tot}$, there will be power left over for recharging the supercapacitor, otherwise the supercapacitor will not be recharged and the system will not be able to run perpetually. However when the charging conditions prevail, the excess power $P_{e}=P_{h}-P_{tot}$ is directed to supercapacitor with capacitance $C_{s}$, and voltage level, *U*, which will have energy storing capacity of $E_{c}=\frac{1}{2}C_{s}U^{2}$ and hence the storage supercapacitor becomes fully charged in

(11) $$t_{c}=E_{c}/P_{e}$$

seconds. Ideally, this energy can then be used totally for communication purposes. However, the constant current discharging (or charging) of a supercapacitor gives a linear decrease (or increase) in the capacitor potential with time [32]. In general, the usable energy is determined by the voltage level which decreases as the energy of a supercapacitor is used and hence the described full energy will not be available. In practice, 50...80 percent of the total supercapacitor energy can be used due to this. Thus, we denote the useful energy by $E_{u}=\eta E_{c}$, where η refers to the fraction of useful energy relative to the maximum theoretical energy (e.g., 0.50...0.80).

In general, diversity schemes may be applied to the system in order to improve the communications performance. Typically gain of several decibels may be obtained, depending on channel conditions. Diversity is especially effective at mitigating multipath situations in indoor or outdoor environments, when no line of sight exist between transmitter and receiver. However, the system power consumption will be increased somewhat due to multiple transmitting and/or receiving units operating simultaneously.

In general, diversity system of order $M_{d}$ will provide theoretical gain of

(12) $$G_{\mathrm{div}}=M_{d}\times \eta_{\mathrm{div}}=M_{r}\times M_{t}\times \eta_{\mathrm{div}},$$

where $M_{r}$ and $M_{t}$ are reception and transmission diversity orders, respectively. The combined efficiency of diversity schemes is $\eta_{\mathrm{div}}$ (≤1), which depends on channel rank and system implementation issues of receiver and transmitter [24]. Due to possible common functionalities for diversity branches, the receiver power consumption $P_{RX}$ due to $M_{r}$-level Maximal Ratio Combining (MRC) diversity functionality may be taken into account by defining

(13) $$P_{RX}=\left(1+\rho_{M}\times \left(M_{r}-1\right)\right)\times P_{RX}^{{}^{\prime }},$$

where $\rho_{M}\leq 1$ is the powerwise efficiency of diversity implementation and $P_{RX}^{{}^{\prime }}$ is the power consumption of individual simple receiver, i.e., without any diversity functionality.

Furthermore, by introducing the power ratio

(14) $$\rho =P_{RX}/P_{TX},$$

where $P_{TX}$ denotes the total power consumption of the transmitter, it is possible to evaluate the system performance more realistically. The overhead power consumption for the peripheral circuitry of the transmitter, ${\hat{P}}_{TX}$, is independent of the transmitter power and diminishes the actual transmit power $P_{TX}$. Thus, the effective transmit power in $M_{t}$ transmit diversity scenario can be related to the power consumed by the transmitter as

(15) $$S_{TX}=M_{t}\times \alpha \left(P_{TX}-{\hat{P}}_{TX}\right),$$

where α≤1 is the transmitter power efficiency. For example, α is close to 0.1 in ZigBee transceivers [33].

### 3.2. Power Consumption and Wake-Up Radio Aspects

**Figure 2:**
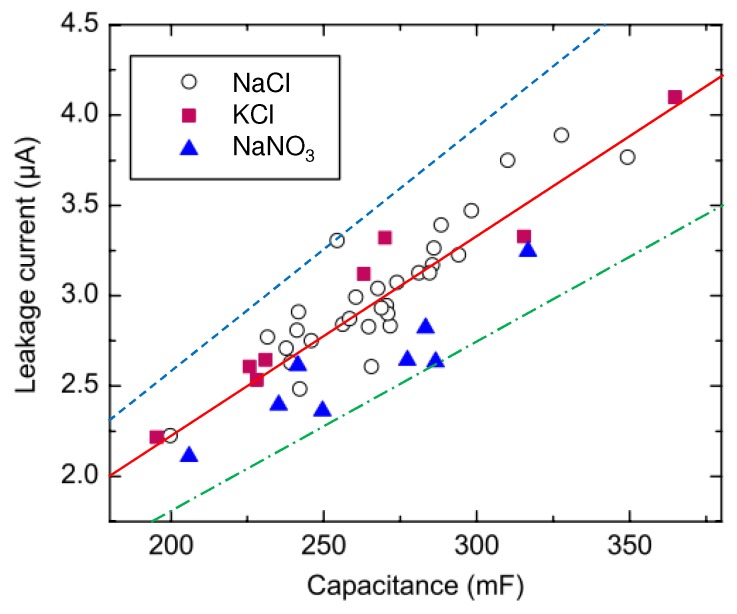
Leakage current behavior with respect to supercapacitor capacitance introduced in [18]. Corresponding median fitted line with $I_{l}\approx 1.11\times 10^{-5}\;C_{s}$ (—), is shown and additionally upper limit behavior $I_{l,\mathrm{up}}\approx 1.31\times 10^{-5}\;C_{s}$ (- - -) and lower limit behavior $I_{l,\mathrm{low}}\approx 0.929\times 10^{-5}\;C_{s}$ (- . - . -) may be seen.

Next we address the achievable operational or communication time using the available harvested energy. Besides using the energy for transmitting and receiving, part of the stored energy is lost due to the self-discharge of the supercapacitor. This can and should be taken into account when calculating the operational time by incorporating supercapacitor leakage current into the analysis. In Figure 2, typical leakage current behavior of several supercapacitors [18] produced by our group is depicted. As shown also by the fitted line, the leakage current increases with respect to capacitance.

Additionally, the required voltage regulator or DC–DC converter [34] controlling and managing the correct operational voltage level, has the loss current $I_{\mathrm{reg}}$, which depends on regulated power *P*, conversion efficiency $\eta_{\mathrm{conv}}$, and voltage level *U*, as

(16) $$I_{\mathrm{reg}}=\frac{P\cdot (1-\eta_{\mathrm{conv}})}{U}.$$

Here, the $\eta_{\mathrm{conv}}$, refs. [35,36,37,38,39,40,41,42,43] may typically have values 0.8 ± 0.1, if the regulator is of switching voltage type. On the other hand, if the regulator is conventional linear type, the efficiency will be 0.4 ± 0.1. For ultra low power systems, the conversion efficiency of a switched-capacitor voltage doubler-based voltage regulator [44], may be used where the power efficiency typically is 63% ($\eta_{\mathrm{conv}}$ = 0.63).

Now, if the transmitter is actively transmitting $100\beta_{TX}$ percent of operational time, receiver is actively receiving $100\beta_{RX}$ percent of operational time, the regulator loss current is denoted by $I_{\mathrm{reg}}$, and the leakage current of capacitor is $I_{l}$, then the total power consumption is given by

(17) $$P_{tot}=P_{TX}\beta_{TX}+P_{RX}\beta_{RX}+UI_{\mathrm{reg}}+UI_{l}.$$

The leakage current, $I_{l}$, [45] may be approximated accurately by

(18) $$I_{l}=k_{c}C_{s},$$

and by applying the least squares curve fitting to the measured results of our supercapacitor implementations [18] yield to parameter values shown in Table 1.

By setting for example, $\beta_{RX}=1-\beta_{TX}$, i.e., the transmitter is on when receiver is off and vice versa, allows for more simple transceiver implementation. Moreover, it can be noticed that the operational time of the transceiver with fully recharged capacitor, denoted here by $t_{op}$, can be expressed by

(19) $$\begin{array}{cc} t_{op}= & E_{u}/P_{tot}\\ = & \eta E_{c}/\left(P_{TX}\beta_{TX}+P_{RX}\left(1-\beta_{TX}\right)+UI_{\mathrm{reg}}+UI_{l}\right). \end{array}$$

However, this is an exceptional case as typically the reloading is supposed to be taking place continuously during the operation of the transceiver. In addition, depending on supercapacitor charging scenario [18], the leakage current may be characterized by

(20) $$\begin{array}{c} I_{l}=k_{a}\left(e^{k_{b}U}-1\right), \end{array}$$

where the $k_{a}$ and $k_{b}$ are determined by applying least squares curve fitting to measured results [18]. The found parameters for different scenarios are given in Table 2. For different supercapacitor charging scenarios the usable time with constant power consumption, *P*, may be given by

(21) $$\begin{array}{ll} t_{op} & =\eta_{conv}E_{u}/\left(\int_{U_{0}}^{U_{1}}k_{a}\left(e^{k_{b}U}-1\right)UdU+P\right)\\ & =\eta_{conv}E_{u}/[k_{a}U_{0}^{2}/2-k_{a}U_{1}^{2}/2\\ & \;+k_{a}(e^{k_{b}U_{0}}\left(k_{b}U_{0}-1\right)/k_{b}^{2}\\ & \;-e^{k_{b}U_{1}}\left(k_{b}U_{1}-1\right)/k_{b}^{2})]. \end{array}$$

**Figure 3:**
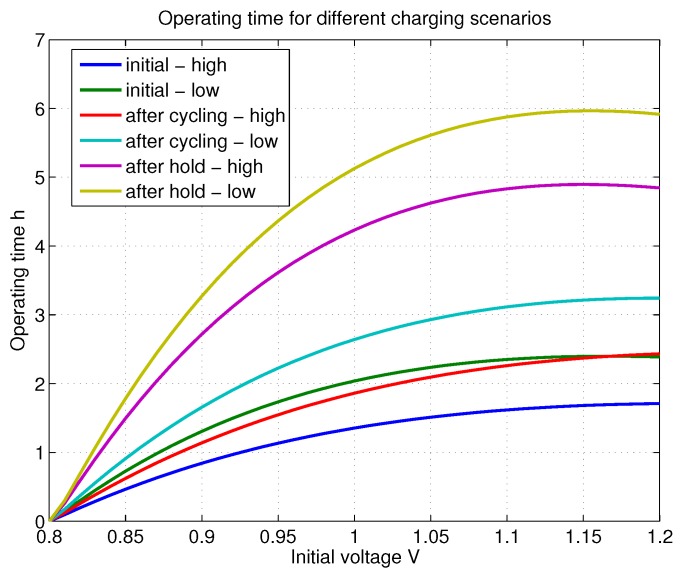
An example illustration of operational times when using different charging scenarios with respect to initial charging voltage level.

Here $U_{1}$ is the initial recharging voltage and $U_{0}$ is the minimum allowed operational voltage level of the system. Next, a small example is given to show how critically the recharging scenario affects the usability. Here, P=−55 dBm, $\eta_{\mathrm{conv}}=0.63$, and η=0.75. The $t_{op}$ with respect to initial voltage in shown in Figure 3. It may be seen that for initial voltage level 1.0 V, the operational times vary between roughly 1.5 h ... 5 h and additionally it is possible to notice that the operating time varies considerably with selected charging scenario.

With traditional simpler IoT systems, the power consumption may be lowered due to small duty cycle provided by the traffic conditions [46]. However, with the constant transceiver operation (17) supposed here, either transmit or receive functioning consumes power. Furthermore, the total power consumption may be lowered and hence operational time increased, by applying energy optimization method based on, e.g., data aware energy efficient distributed clustering protocol, saving cluster head selection energy [47] or applying clustering protocol with genetic algorithm in order to decrease energy consumption [48,49]. To simplify analysis, here it is assumed that no energy optimization algorithms are applied. This will provide fair comparison between the selected scenarios.

However, wake-up radio (WuR) concept is used here, where the whole transceiver can be set to sleep mode and only awakened by the Host node when needed. In the node, special WuR circuitry controls the wake-up process. There are plenty of WuR systems proposed, e.g., in refs. [26,28,50,51,52,53,54]. Most of these apply relatively complex approaches, i.e., use combination of several frequencies to select particular node with WuR for the wake-up, or low power listening mode for WuR to activate the waking up process properly, e.g., at several stages. However, all these approaches are, in some degree, active methods as they consume extra power, and hence are out of our interest. It is evident, that only the fully passive wake-up process would yield to ultimately low power consumption of the system.

**Figure 4:**
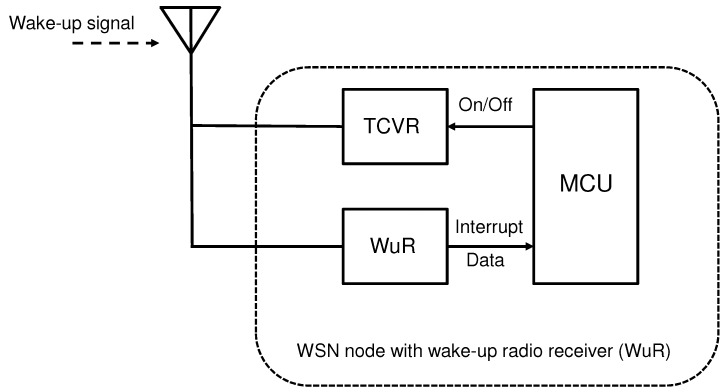
Wake-up Radio (WuR) within wireless sensor node (WSN).

The general concept is shown in Figure 4 where the WuR is controlling the transceiver on/off state by the Microcontroller Unit, MCU. When wake-up state is received, the WuR triggers transceiver on, otherwise it is in off state hence allowing potentially considerable power savings. The system described in the figure, uses the same antenna and frequency bands for the wake-up and transceiver operations. As a result of relatively simple non-selective passive wake-up radio circuitry, false wake-ups may take place. This is more probable to occur when the number of nodes in the system is large and hence the expected wake-up activity increases. Furthermore, even external RF interference may cause false wake-ups in the system. Taking these aspects into account and by supposing that the MCU consumes negligible power, the total power consumption for the passive WuR assisted communication, $P_{wtot}$, may be written as

(22) $$P_{wtot}=\left(\beta_{wTX}P_{TX}+\beta_{wRX}P_{RX}+UI_{\mathrm{reg}}\right)\left(\beta_{w}+\beta_{f}\right)+UI_{l},$$

where $\beta_{wTX}$, $\beta_{wRX}$, $\beta_{w}$, and $\beta_{f}$ are the transmit operation probability, receive operation probability, wake-up operation probability, and false wake-up detection probability due to external interference etc., correspondingly. When the host duty cycle per node is $\beta_{w}^{{}^{\prime }}$ and $N_{no}$ is the number of nodes, i.e., sensors in the system, the wake-up operation probability can be expressed as

(23) $$\beta_{w}=\beta_{w}^{{}^{\prime }}N_{no}.$$

As can be seen, as the total number of nodes increases, number of wake-ups increases and hence the total power consumption is also increased. However, with relatively small $\beta_{w}^{{}^{\prime }}$ and therefore small $\beta_{w}$, the increased power consumption is not critical. This will be illustrated in more details in Section 4, where evaluations of the presented concept will be given.

To improve the passive wake-up radio operation, antennas with relatively high gains might be needed to be used. To keep the performance sufficient, physical sizes feasible [55] and the directivity more controllable, antenna gains that are less than 15 dBi might be preferable. The high gain antenna may be located at the wake-up transmitter to allow simpler node implementation with smaller gain node antenna. By defining $S_{wTX}$ to be the transmitted wake-up signal power, the received wake-up signal power is given by

(24) $$S_{wRX}=G_{a}S_{wTX}/\lambda_{w},$$

where $G_{a}$ is the combined transmit-receive antenna gain and $\lambda_{w}$ denotes the path loss between wake-up transmitter and wake-up receiver, i.e., within wake-up distance, $d_{w}$.

It turns out that with passive wake-up circuitry the operational range is quite limited, as the wake-up operation needs to collect the required energy from the wake-up signal transmitted by the host [26,27]. In addition latency will take place, but in practice it will be in the order of tens of milliseconds and is most often tolerable, considering the normal use cases, especially in low bitrate use cases.

To control and limit the false wake-ups better and hence avoiding the increase of power consumption of the node, semi-passive wake-up radio concept may be introduced. Here the wake-up radio is not fully passive, but a small amount of power is consumed by the low complexity wake-up circuitry so that the wake-up command can be decoded accurately in hibernation mode as, e.g., in Ref. [50]. The system might be designed so that the hibernation mode power, $P_{hib}$, is just a fraction of $P_{RX}$, and for later use this fraction can be defined as

(25) $$\rho_{sw}=P_{hib}/P_{RX}.$$

Taking these aspects into account and by supposing again that the MCU consumes negligible power, the total power consumption for the semi-passive WuR assisted communication, $P_{swtot}$, may be written as

(26) $$\begin{array}{ll} P_{swtot}= & \left(\beta_{wTX}P_{TX}+\beta_{wRX}P_{RX}+UI_{\mathrm{reg}}\right)\beta_{w}^{{}^{\prime }}\\ & +P_{hib}\left(1-\beta_{w}^{{}^{\prime }}\right)+UI_{l}, \end{array}$$

where in addition to the previously defined variables, power $P_{hib}$ is introduced, modeling the power consumption of wake-up radio receiver in hibernation mode. As can be seen, when the host activity $\beta_{w}^{{}^{\prime }}$ is small, $P_{hib}\left(1-\beta_{w}^{{}^{\prime }}\right)$ determines the power consumption of the transceiver operation. Thus for relatively low activity situations, remarkable power consumption reductions may be expected especially when additionally $P_{hib}$ is small. Furthermore, the latency is decreased considerably as there is no need for capacitor reloading as in fully passive wake-up system.

The issues addressed here will be studied and evaluated through numerical examples in the following section.

## 4. Results and Analysis

In this section, the considered channel models are first briefly introduced. After that, example use case is presented along with some illustrative evaluations. The focus will be on low energy, low bit rate, robust, and self sufficient system, using energy harvesting. In general, energy harvesting and wake-up process have not effect on QoS parameters (e.g., end-to-end latency, throughput and packet loss ratio) in the proposed case.

### 4.1. Considered Pathloss Models and Use Cases

Here, IEEE 802.11ah channel models [56] are used to model a multitude of M2M communication scenarios, incorporating outdoor with macro, outdoor pico/hotzone deployments, indoor, and outdoor Device to Device (D2D) use cases as shown in Table 3. In outdoor macro antenna height is assumed 15 m above rooftop, whereas in pico/hotzone deployments antenna is assumed at rooftop level. For outdoor D2D path loss, antenna height is assumed 1.5 m. In indoor path loss case, the model is valid for single floor scenario and the exact antenna height is not specified, but can be typically in practise assumed to be less than 3 m. The typical path lengths for the models are some hundreds of meters, but for macro channel even several kilometers. For other center frequencies, *f*, a correction term of $21\log_{10}(f/$900 MHz) should be added. For completeness, the Free Space Loss attenuation [57] is given by

(27) $$L_{\mathrm{FSL}}\left(d\right)=20\log_{10}\left(d\right)+20\log_{10}\left(f_{c}\right)-27.55.$$

Notice that even though the 802.11ah system itself is assumed to be deployed only at the 900 MHz (sub-1 GHz) band, the above path loss models are indeed valid at other frequencies as well, as long as the proper correction term stated earlier is applied.

### 4.2. Capacity Evaluations

**Figure 5:**
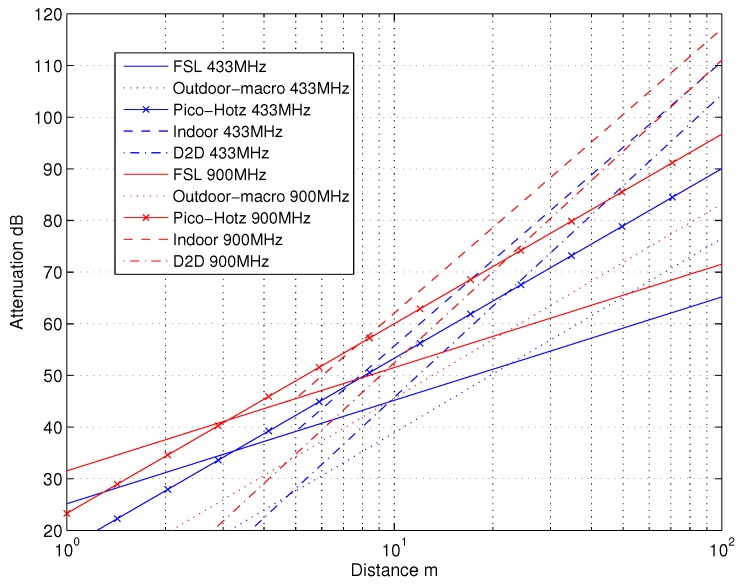
Path losses for considered 802.11ah channels at 433 MHz and 900 MHz bands. Here $d_{BP}=5$ for indoor channel. Free space loss, FSL, is also shown for reference.

**Figure 6:**
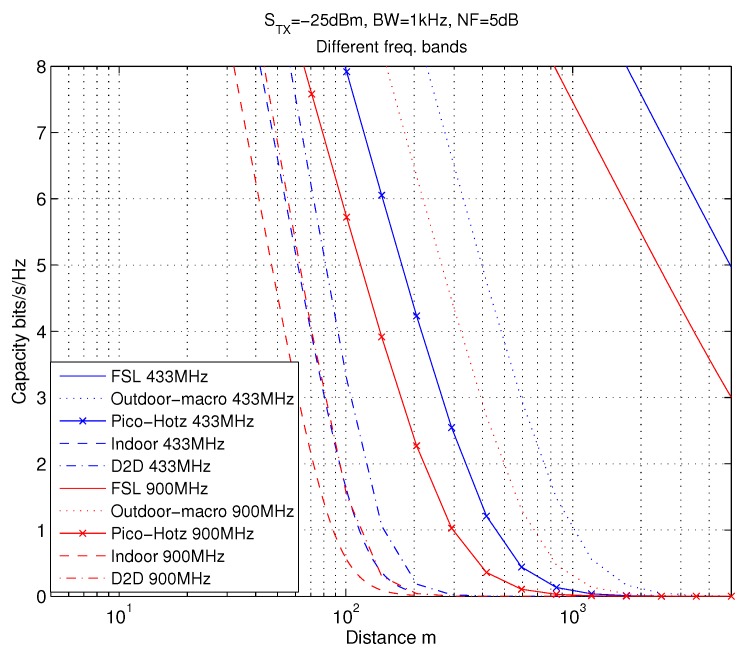
Relative capacity for 802.11ah channels at 433 MHz and 900 MHz bands. Here $d_{BP}=5$ for indoor channel. $S_{TX}=-25$ dBm, B= 1 kHz, T = 290 K, and NF = 5 dB.

Here, in the numerical evaluations, we specifically focus on the license-exempt ISM bands at 433 MHz and 900 MHz (sub-1GHz), due to their good suitability for low-power communications and being free from spectrum licensing related constraints. The lighting level is assumed to be as defined for working and processing environment (rough assembly) [58], i.e., 200 lx . The principal path loss behaviors in the considered use cases versus the communication distance, *d*, are illustrated in Figure 5 at these two frequency bands. The path loss of indoor channel A with $d_{BP}=5$ shown in the figure, is the most demanding channel in path loss sense. Thus, this channel case will be considered in the continuation for path loss modeling. Moreover, the capacity (3) can be evaluated for the considered channels. As can be seen in Figure 6, e.g., in Indoor A channel when $S_{TX}$ = −25 dBm and the capacity requirement is 2 bits/s/Hz, the maximum communication distance is 70 m at 900 MHz. For 433 MHz the distance will be increased by roughly 20 m, in this case. Similarly for 1 bit/s/Hz capacity, the distances will be 90 m and 110 m, correspondingly.

In the following example scenario, the critical parameters for evaluations are collected into Table 4.

**Figure 7:**
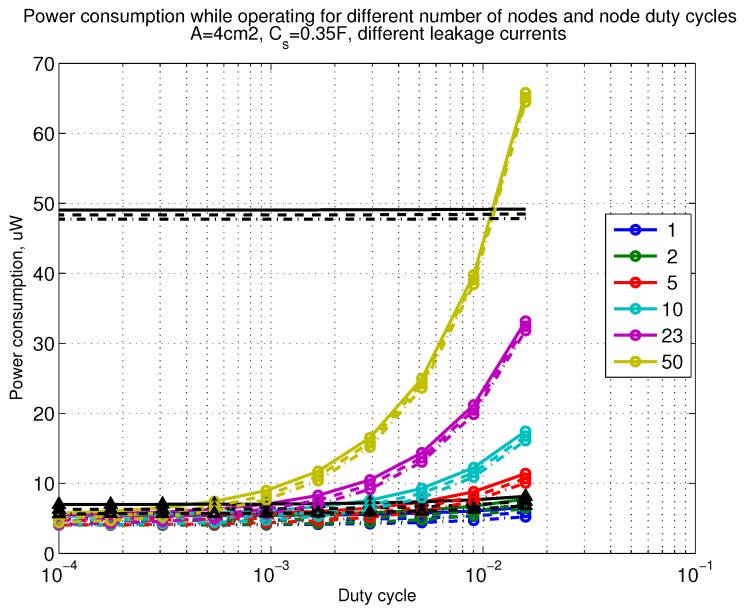
When $M_{t}=1$ and $M_{r}=1$, power consumption while operating with reloading the capacitor while operating with capacitor ($C_{s}=0.35$ F). Non-WuR in black (without triangles), passive WuR in colors indicating number of nodes for different host duty cycles per node ($\beta_{w}^{{}^{\prime }}$). Semi-passive WuR in black with triangles. Line type indicates used SC leakage current model as —: upper , - - -: median, and -.-.-: lower.

**Figure 8:**
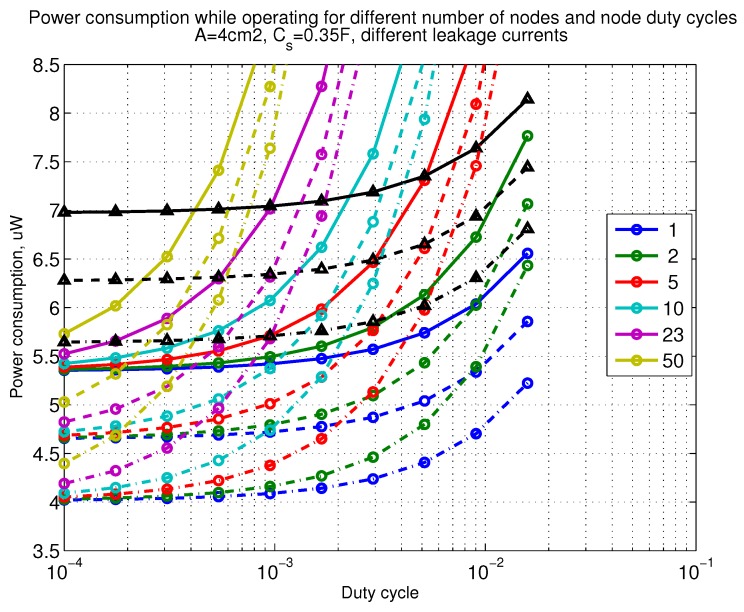
Detailed figure when $M_{t}=1$ and $M_{r}=1$, power consumption while operating with reloading the capacitor while operating with capacitor ($C_{s}=0.35$ F). Non-WuR in black (without triangles), passive WuR in colors indicating number of nodes for different host duty cycles per node ($\beta_{w}^{{}^{\prime }}$). Semi-passive WuR in black with triangles. Line type indicates used SC leakage current model as —: upper , - - -: median, and -.-.-: lower.

**Figure 9:**
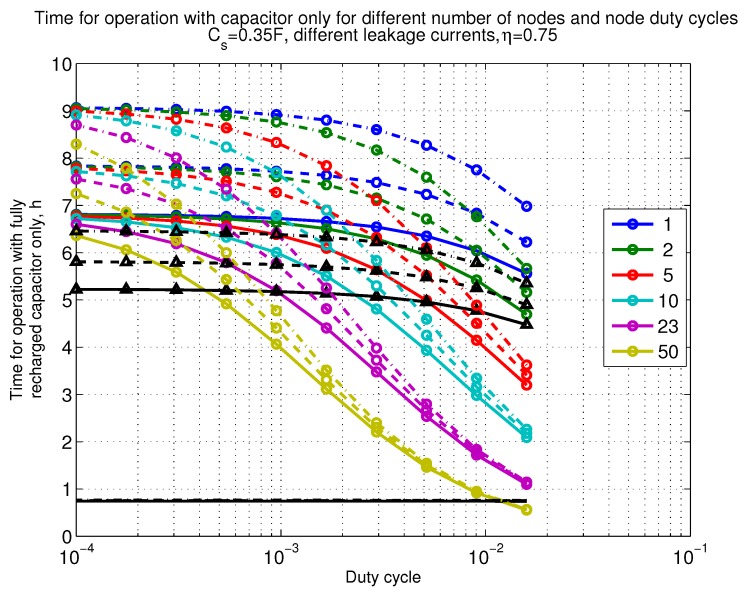
When $M_{t}=1$ and $M_{r}=1$, time for operation with capacitor ($C_{s}=0.35$ F) only. Non-WuR in black (without triangles), passive WuR in colors indicating number of nodes for different host duty cycles per node ($\beta_{w}^{{}^{\prime }}$). Semi-passive WuR in black with triangles. Line type indicates used SC leakage current model as —: upper , - - -: median, and -.-.-: lower.

**Figure 10:**
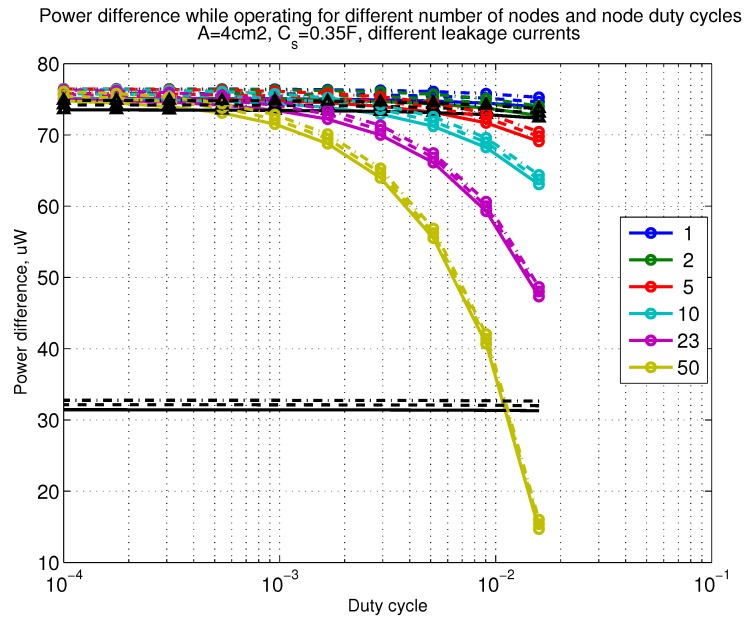
When $M_{t}=1$ and $M_{r}=1$, power difference while operating. Capacitor ($C_{s}=0.35$ F). Non-WuR in black (without triangles), passive WuR in colors indicating number of nodes for different host duty cycles per node ($\beta_{w}^{{}^{\prime }}$). Semi-passive WuR in black with triangles. Line type indicates used SC leakage current model as —: upper , - - -: median, and -.-.-: lower.

**Figure 11:**
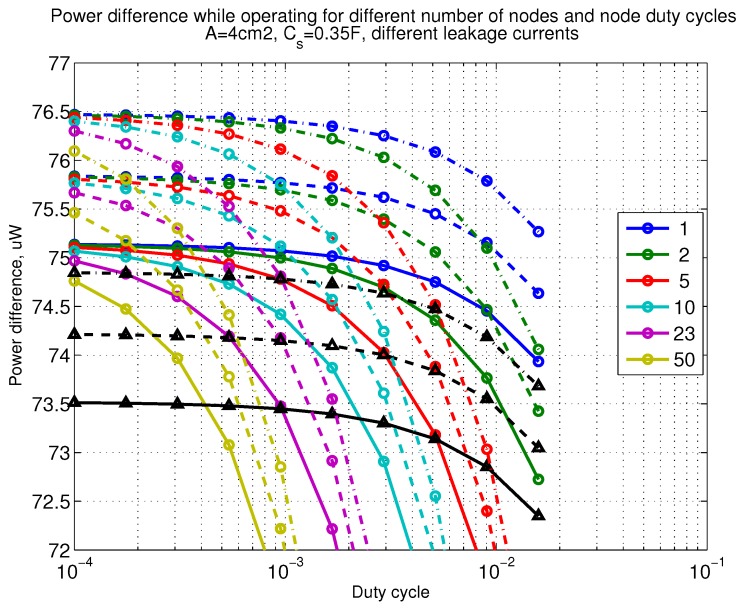
Detailed figure when $M_{t}=1$ and $M_{r}=1$, power difference while operating. Capacitor ($C_{s}=0.35$ F). Non-WuR in black (without triangles), passive WuR in colors indicating number of nodes for different host duty cycles per node ($\beta_{w}^{{}^{\prime }}$). Semi-passive WuR in black with triangles. Line type indicates used SC leakage current model as —: upper , - - -: median, and -.-.-: lower.

**Figure 12:**
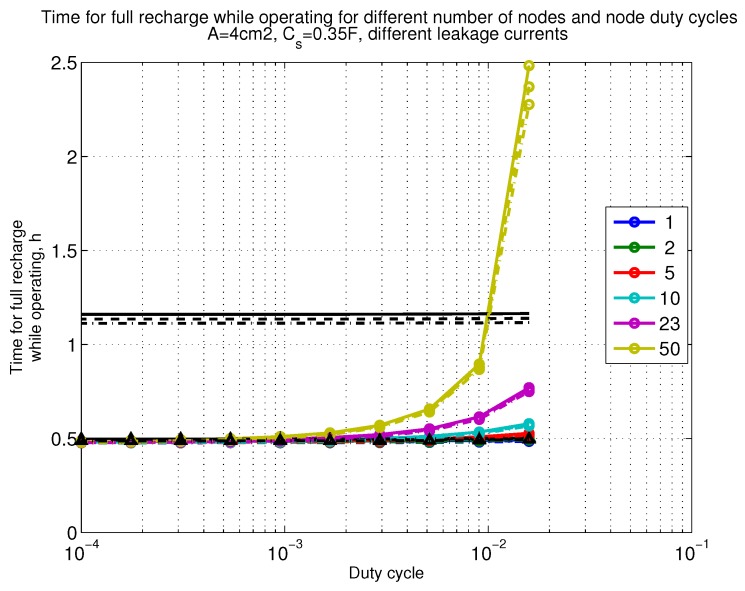
When $M_{t}=1$ and $M_{r}=1$, time for full recharge while operating with capacitor ($C_{s}=0.35$ F). Non-WuR in black (without triangles), passive WuR in colors indicating number of nodes for different host duty cycles per node ($\beta_{w}^{{}^{\prime }}$). Semi-passive WuR in black with triangles. Line type indicates used SC leakage current model as —: upper, - - -: median , and -.-.-: lower.

**Figure 13:**
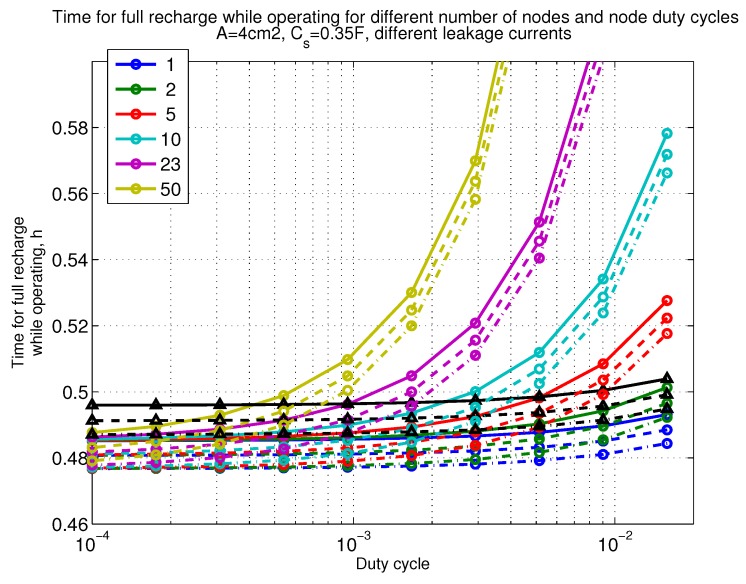
Detailed figure when $M_{t}=1$ and $M_{r}=1$, time for full recharge while operating with capacitor ($C_{s}=0.35$ F). Non-WuR in black (without triangles), passive WuR in colors indicating number of nodes for different host duty cycles per node ($\beta_{w}^{{}^{\prime }}$). Semi-passive WuR in black with triangles. Line type indicates used SC leakage current model as —: upper , - - -: median, and -.-.-: lower.

The power consumption of different communication scenarios without diversity, i.e., $M_{t}=1$ and $M_{r}=1$ is shown in Figure 7. As can be noticed, the system without WuR assistance consumes almost constantly the largest amount of power. However when $\beta_{w}^{{}^{\prime }}>10^{-2}$, the system with 50 nodes starts to consume more than the system without WuR due to false wake-ups. More detailed view on power consumption using WuR methods is shown in Figure 8. Time for operation with SC only is shown in Figure 9, where the WuR systems are providing five times more operational time than non-WuR approaches in low duty cycle cases. The power difference between the harvested power and the consumed power is evaluated in Figure 10, where it can be seen that for the low duty cycle values the power accumulation is more than two times larger for WuR assisted cases than in non-WuR case. More detailed view on power difference may be seen in Figure 11. The time for full recharge while operating may be seen in Figure 12 and with more details for WuR cases in Figure 13.

**Figure 14:**
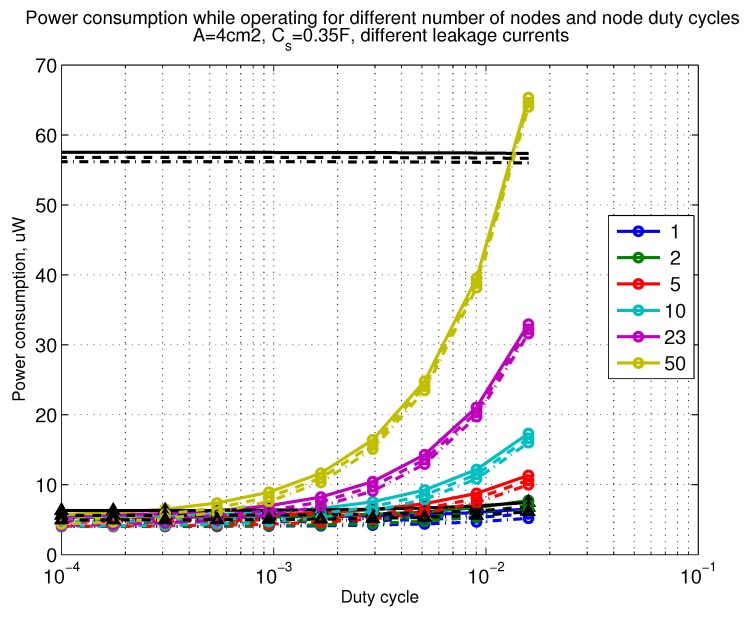
When $M_{t}=1$ and $M_{r}=2$, power consumption while operating with reloading the capacitor while operating with capacitor ($C_{s}=0.35$ F). Non-WuR in black (without triangles), passive WuR in colors indicating number of nodes for different host duty cycles per node ($\beta_{w}^{{}^{\prime }}$). Semi-passive WuR in black with triangles. Line type indicates used SC leakage current model as —: upper , - - -: median, and -.-.-: lower.

**Figure 15:**
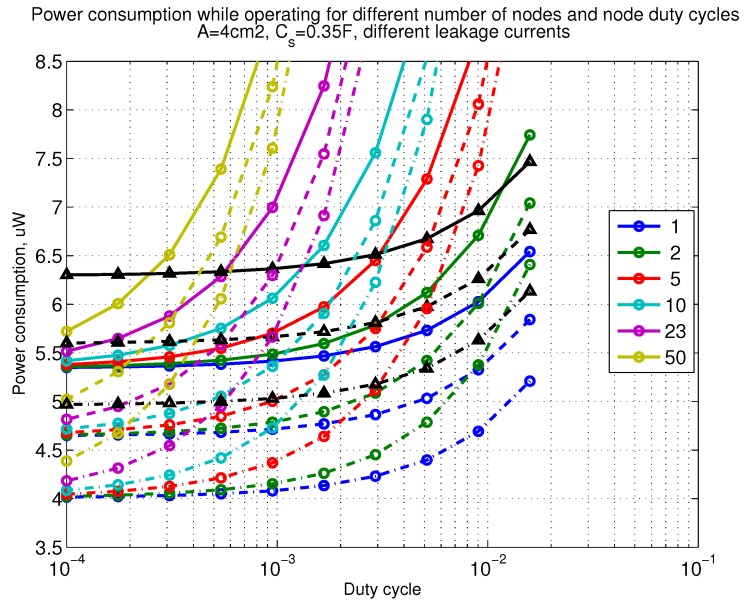
Detailed figure when $M_{t}=1$ and $M_{r}=2$, power consumption while operating with reloading the capacitor while operating with capacitor ($C_{s}=0.35$ F). Non-WuR in black (without triangles), passive WuR in colors indicating number of nodes for different host duty cycles per node ($\beta_{w}^{{}^{\prime }}$). Semi-passive WuR in black with triangles. Line type indicates used SC leakage current model as —: upper , - - -: median, and -.-.-: lower.

**Figure 16:**
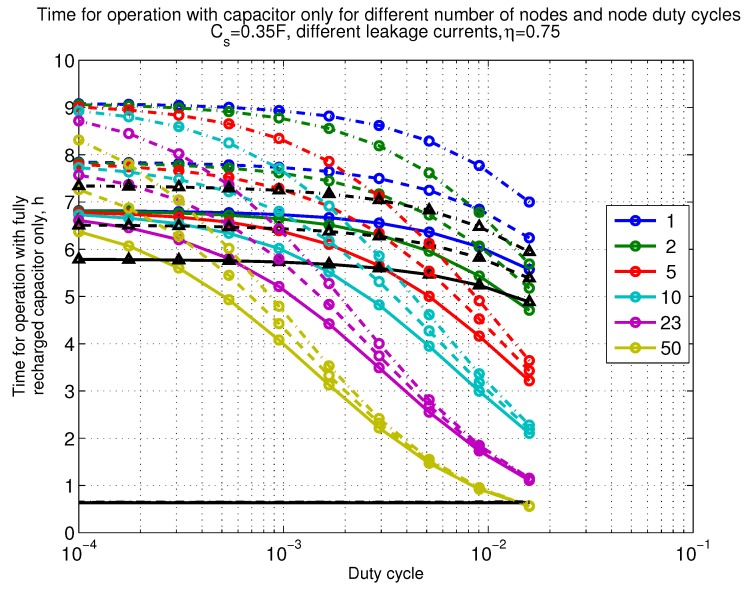
When $M_{t}=1$ and $M_{r}=2$, time for operation with capacitor ($C_{s}=0.35$ F) only. Non-WuR in black (without triangles), passive WuR in colors indicating number of nodes for different host duty cycles per node ($\beta_{w}^{{}^{\prime }}$). Semi-passive WuR in black with triangles. Line type indicates used SC leakage current model as —: upper , - - -: median, and -.-.-: lower.

**Figure 17:**
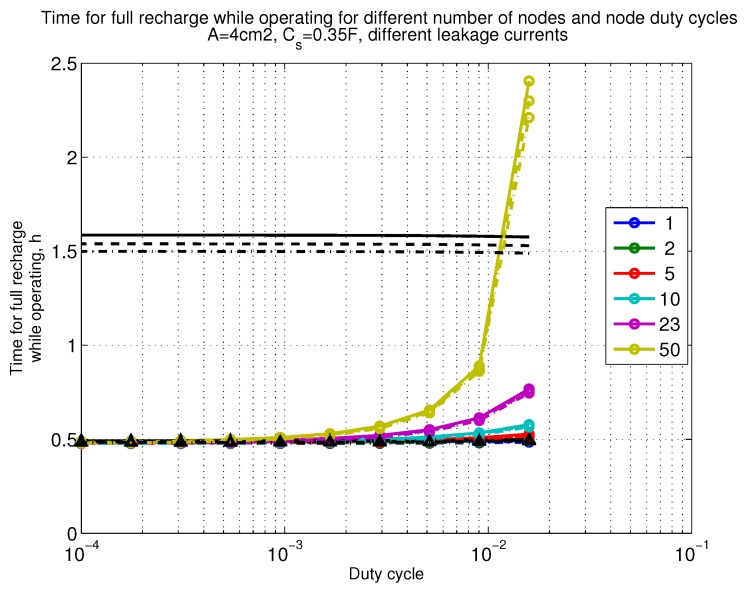
When $M_{t}=1$ and $M_{r}=2$, time for full recharge while operating with capacitor ($C_{s}=0.35$ F). Non-WuR in black (without triangles), passive WuR in colors indicating number of nodes for different host duty cycles per node ($\beta_{w}^{{}^{\prime }}$). Semi-passive WuR in black with triangles. Line type indicates used SC leakage current model as —: upper, - - -: median , and -.-.-: lower.

**Figure 18:**
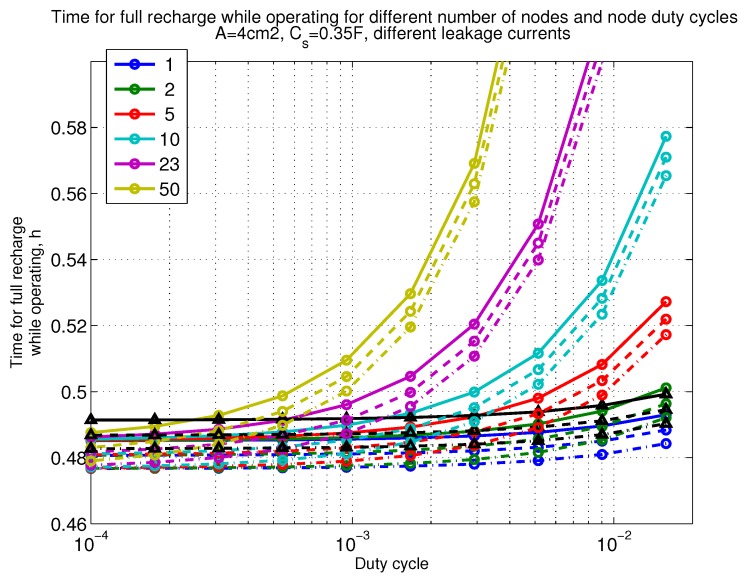
Detailed figure when $M_{t}=1$ and $M_{r}=2$, time for full recharge while operating with capacitor ($C_{s}=0.35$ F). Non-WuR in black (without triangles), passive WuR in colors indicating number of nodes for different host duty cycles per node ($\beta_{w}^{{}^{\prime }}$). Semi-passive WuR in black with triangles. Line type indicates used SC leakage current model as —: upper , - - -: median, and -.-.-: lower.

**Figure 19:**
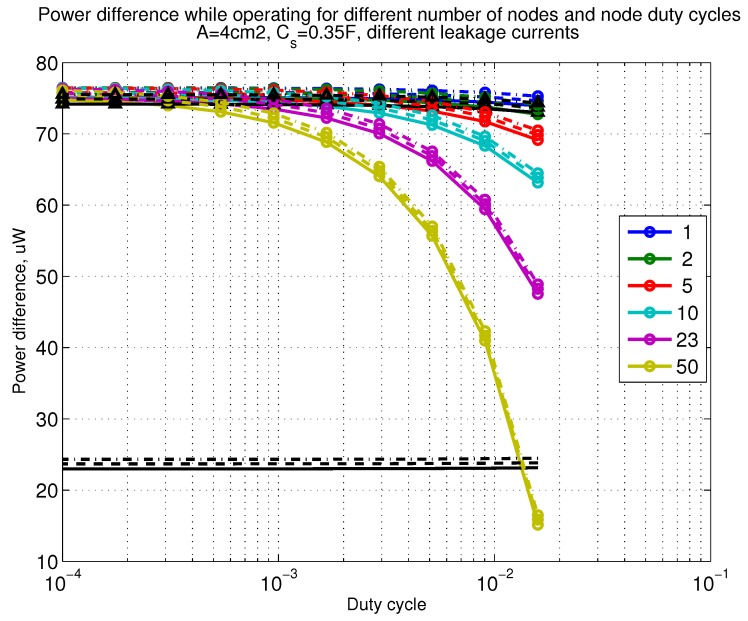
When $M_{t}=1$ and $M_{r}=2$, power difference while operating. Capacitor ($C_{s}=0.35$ F). Non-WuR in black (without triangles), passive WuR in colors indicating number of nodes for different host duty cycles per node ($\beta_{w}^{{}^{\prime }}$). Semi-passive WuR in black with triangles. Line type indicates used SC leakage current model as —: upper , - - -: median, and -.-.-: lower.

**Figure 20:**
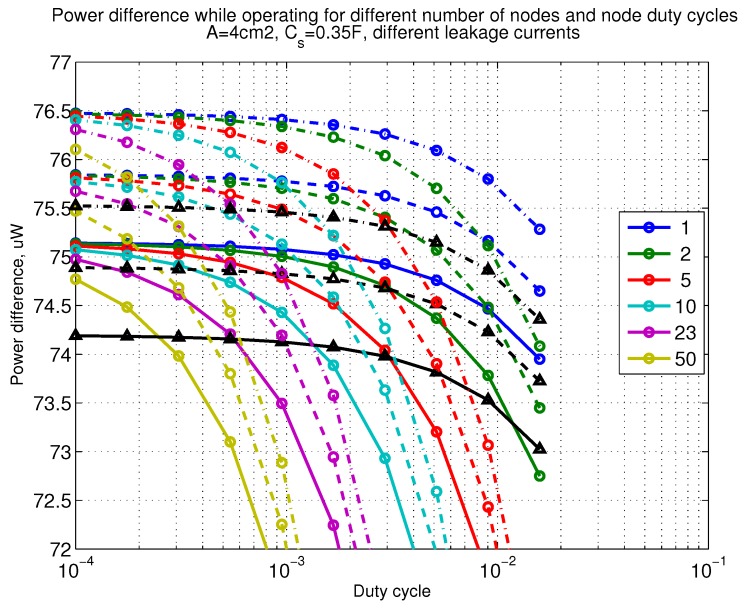
Detailed figure when $M_{t}=1$ and $M_{r}=2$, power difference while operating. Capacitor ($C_{s}=0.35$ F). Non-WuR in black (without triangles), passive WuR in colors indicating number of nodes for different host duty cycles per node ($\beta_{w}^{{}^{\prime }}$). Semi-passive WuR in black with triangles. Line type indicates used SC leakage current model as —: upper , - - -: median, and -.-.-: lower.

To complete the collection of evaluations, the results are given in the Figure 14, Figure 15, Figure 16, Figure 17, Figure 18, Figure 19 and Figure 20 for diversity case with $M_{t}=1$ and $M_{r}=2$. By comparing power consumption results in Figure 7 and Figure 8 with corresponding diversity results shown in Figure 14 and Figure 15, it is possible to see that the example diversity system, with selected practical parameters, uses slightly smaller power for the same performance. Here, the small performance difference is due to relatively large implementation losses in the considered diversity scenario. Similar kind of findings and likenesses may be found in the remaining Figure 16, Figure 17, Figure 18, Figure 19 and Figure 20, when comparing with non-diversity Figure 7, Figure 8, Figure 9, Figure 10 and Figure 11.

## 5. Conclusions

In this paper, the limits on wireless energy-harvesting and related communications were studied and evaluated. In the analysis, elementary path loss models and implementation losses were included. Additionally, diversity system gains and implementation losses were included. Moreover, wireless RF and photovoltaic energy harvesting and semi-passive and passive wake-up radio concepts with realistic design parameters were included in the study. Furthermore, as a practical example, a state-of-the-art printed supercapacitor model with leakage current properties was adopted to store the energy scavenged and additionally DC/DC conversion losses were incorporated. A lSarge set of numerical results were given, specifically at the license-exempt sub-1GHz ISM bands, while the presented methodology and reported results will be usable also at other frequencies. The provided analysis principles and outcomes establish clear feasibility and performance bounds for wireless energy harvesting based low bitrate M2M communications in the future IoT networks. 

## Figures and Tables

**Table 1:** Leakage current model parameters based on results given in [18].

Supercapacitor Type	Coefficient for Lower Limit, $k_{c}$	Coefficient for Upper Limit, $k_{c}$
NaCl	0.973 × 10${}^{-5}$	1.31 × 10${}^{-5}$
KCl	1.07 × 10${}^{-5}$	1.22 × 10${}^{-5}$
NaNO_3_	0.929 × 10${}^{-5}$	1.09 × 10${}^{-5}$

**Table 2:** Calculated leakage current model parameters for different recharging scenarios based on results given in [18].

Recharging Scenario	Coefficient $k_{a}$	Coefficient $k_{b}$
Initial—high	4.74 × 10${}^{-7}$	2.70
Initial—low	1.71 × 10${}^{-7}$	3.32
After cycling—high	4.64 × 10${}^{-7}$	2.40
After cycling—low	1.88 × 10${}^{-7}$	2.95
After hold—high	6.69 × 10${}^{-8}$	3.53
After hold—low	5.78 × 10${}^{-8}$	3.48

**Table 3:** Propagation loss in decibels for different channels [56].

Channel	Propagation Loss, $L_{\mathrm{Channel}}\left(d\right)$, *d* in Meters and $f_{c}$ Is 900 MHz
Outdoor-macro	8 + 37.6$\log_{10}\left(d\right)$
Pico-hotzone	23.3 + 36.7$\log_{10}\left(d\right)$
D2D	58.6$\log_{10}\left(d\right)$-6.17
Indoor	20$\log_{10}\left(4\pi df_{c}/c\right)$ $(=L_{FS}\left(d\right)),\mathrm{for}d\leq d_{BP}$
	$L_{FS}\left(d_{BP}\right)+35\log_{10}\left(d/d_{BP}\right)$, for $d>d_{BP}$
	$d_{BP}=\left\{5,5,5,10,20,30\right\}$ [m] are
	breakpoint distances for A...F models, correspondingly.

**Table 4:** Parameter values for simulations.

Parameter	Value(s)	Note(s)
Indoor A channel	$d_{BP}=5$ m	Breakpoint distance
*B*	1 kHz	
*C*	1 bit/s/Hz	→ R = 1 kbit/s
*T*	290 K	
NF	5 dB	
$P_{TX}$	−25 dBm	
${\hat{P}}_{TX}$	−37 dBm	
$C_{s}$	0.35F	
α	0.1	
ρ	0.75	$P_{RX}/P_{TX}$
$\rho_{sw}$	0.10	$P_{hib}/P_{RX}$
μ	3.16 (5dB)	
$\eta_{\mathrm{div}}$	0.7	
$\rho_{M}$	0.9	
*U*	1 V	
$I_{l}$	$k_{c}$ × 10${}^{-5}$ $C_{s}$	
η	0.75 (75%)	
$\eta_{\mathrm{conv}}$	0.63 (63%)	[44]
$\beta_{wTX}$	1	
$\beta_{wRX}$	1	
$\beta_{w}^{{}^{\prime }}$	10${}^{-4}$ ... 1.6 × 10${}^{-2}$	$=\beta_{TX}$
$\beta_{f}$	0.01	
$N_{no}$	1, 2, 5, 10, 23, and 50	
$G_{h}$	1 (0 dBi)	
$\eta_{h}$	0.40	
$P_{htx}$	10 dBm @ 433 MHz	
*A*	4 cm${}^{2}$	
*E*	2 W/m${}^{2}$	200 lx [58]
$\eta_{c}$	0.10 (10%)	
$S_{wTX}$	10 dBm @ 433 MHz	
$G_{a}$	10 dBi	
